# The use of green tea polyphenols for treating residual albuminuria in diabetic nephropathy: A double-blind randomised clinical trial

**DOI:** 10.1038/srep28282

**Published:** 2016-06-20

**Authors:** Cynthia M. Borges, Alexandros Papadimitriou, Diego A. Duarte, Jacqueline M. Lopes de Faria, José B. Lopes de Faria

**Affiliations:** 1 Renal Pathophysiology Laboratory, Investigation on Diabetes Complications, Nephrology Unit, Faculty of Medical Sciences (FCM), State University of Campinas (UNICAMP), Campinas, São Paulo, Brazil

## Abstract

Prior research has shown that in experimental diabetes mellitus, green tea reduces albuminuria by decreasing podocyte apoptosis through activation of the WNT pathway. We investigated the effect of green tea polyphenols (GTP) on residual albuminuria of diabetic subjects with nephropathy. We conducted a randomised, double-blind study in 42 diabetic subjects with a urinary albumin-creatinine ratio (UACR) >30 mg/g, despite administration of the maximum recommended dose of renin-angiotensin (RAS) inhibition. Patients were randomly assigned to two equal groups to receive either GTP (containing 800 mg of epigallocatechin gallate, 17 with type 2 diabetes and 4 with type 1 diabetes) or placebo (21 with type 2 diabetes) for 12 weeks. Treatment with GTP reduced UACR by 41%, while the placebo group saw a 2% increase in UACR (*p* = 0.019). Podocyte apoptosis (*p* = 0.001) and *in vitro* albumin permeability (*p* < 0.001) were higher in immortalized human podocytes exposed to plasma from diabetic subjects compared to podocytes treated with plasma from normal individuals. In conclusion, GTP administration reduces albuminuria in diabetic patients receiving the maximum recommended dose of RAS. Reduction in podocyte apoptosis by activation of the WNT pathway may have contributed to this effect.

Multifactorial treatment by means of blood glucose and blood pressure control, including maximum doses of angiotensin-converting enzyme (ACE) inhibitors or angiotensin receptor blockers (ARBs), as well as administration of lipid lowering drugs, is an efficient, though insufficient, approach to treatment for diabetic nephropathy (DN)^1^. Despite the availability of so many effective interventions, DN remains the main cause of end-stage renal disease in most parts of the world^2^.

In patients with DN, post hoc analyses of trial outcomes have demonstrated a robust relationship between the magnitude of albuminuria reduction and the slowing of chronic kidney disease (CKD) and reduced rates of cardiovascular events^3^,^4^,^5^,^6^,^7^,^8^. In addition, the high residual risk in diabetic nephropathy is directly related to the residual albuminuria in these patients^9^. Therefore, new treatment options must be added to the current armamentarium, especially drugs that can lower residual risk factors without increasing adverse events.

Preclinical studies in diabetic nephropathy^10^,^11^,^12^ and in rapidly progressive glomerulonephritis^13^ have demonstrated that green tea (GT) is able to reduce albuminuria and other markers of renal damage. In experimental DN studies, GT was able to reduce oxidative stress markers by inhibiting NOX4, as well as nitric oxidase synthase (NOS) uncoupling^10^,^11^. In addition, we recently demonstrated that under experimental diabetic conditions, GT reduces podocyte apoptosis by activating the WNT pathway^12^. Interestingly, GT was not able to reduce podocyte apoptosis when podocytes exposed to high glucose were treated with dickkopf 1 (DKK-1). DKK-1 is a blocker of low-density lipoprotein receptor-related protein 6 (LRP6), a receptor of the WNT pathway that is involved in podocyte death under conditions of high glucose exposure^14^. We concluded that GT could ameliorate albuminuria by reducing podocyte apoptosis through activation of the WNT pathway^12^.

A recent meta-analysis of studies in human subjects concluded that moderate consumption of GT reduces the risk of cardiovascular events and stroke by enhancing endothelial-dependent vasodilation^15^. In addition, the use of the main GT catechin, epigallocatechin gallate (EGCG), administered in doses of up to 800 mg daily, is safe for healthy postmenopausal women and reduces their LDL-cholesterol and plasma insulin levels^16^. To our knowledge, no study in humans has evaluated the potential efficacy of GT in treating patients with DN.

The aim of this study was to investigate whether green tea polyphenols (GTP) can reduce residual albuminuria in diabetic subjects receiving the maximum recommended dose of ACE inhibitors and/or ARBs and to test the hypothesis that GTP exerts its antiproteinuric effect via reduction of podocyte apoptosis by activating the WNT pathway.

## Results

**Figure 1:**
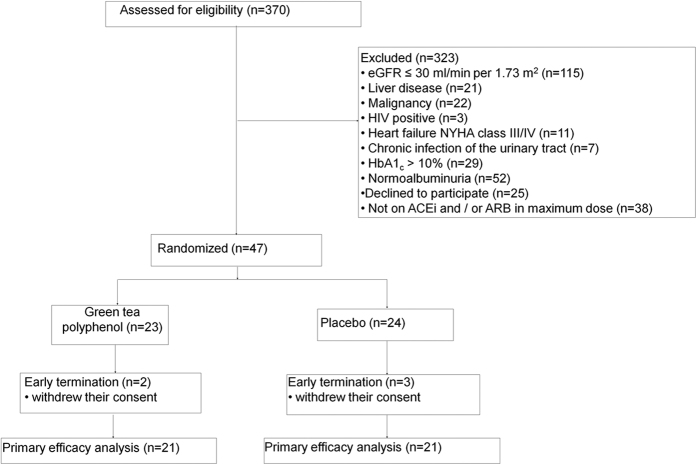
Flow diagram of the trial. eGFR, estimated glomerular filtration rate; HIV, human immunodeficiency virus; NYHA, New York Heart Association; HbA1c, glycated hemoglobin; ACEi, angiotensin-converting enzyme inhibitor; ARB, angiotensin receptor blocker.

The screening phase of this trial started in November 2013; the treatment phase was completed by December 2014. As shown in the flow diagram (Fig. 1), 47 patients were enrolled in the study and randomly assigned to one of two groups to receive either GTP or placebo (Table 1). Two patients in the GTP group and three patients in the placebo group were ultimately excluded from final analysis of the study due to withdrawal of their consent and unavailability of final laboratory data. Eventually, 21 patients in each group completed the 12-week treatment phase of the trial.

### Primary endpoint

**Figure 2:**
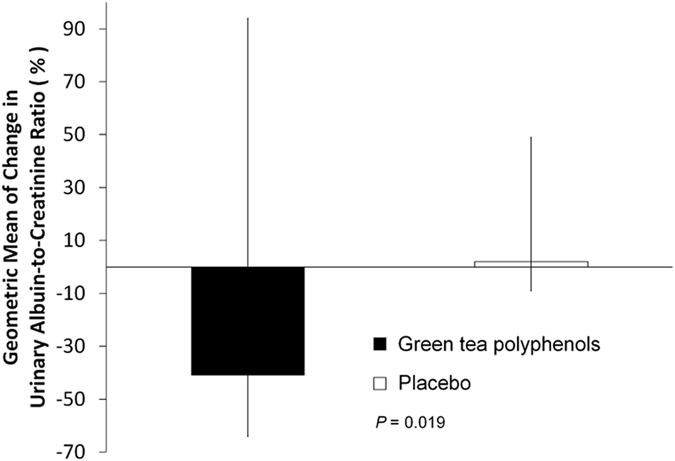
Patients in the green tea polyphenol group (n = 21) experienced a significant reduction in UACR, while patients in the placebo (n = 21) group experienced a small increase. Geometric mean of % change in urinary albumin-creatinine ratio from baseline to the end of the study. Vertical bars represent the 95% confidence intervals. *P* value is for comparison between the green tea polyphenol and the placebo groups.

The same regime reported at baseline for RAS inhibition was maintained in all 42 patients who completed the study. Patients receiving GTP had a median (range) UACR of 210 mg/g (39–1267) at baseline, which was reduced to 133 mg/g (10–1812). Patients receiving placebo, on the other hand, experienced an increase in UACR, from 427 mg/g (77–4051) at baseline to 452 mg/g (43–4802) by week 12. After 12 weeks of treatment, GTP had reduced the geometric mean of % change from baseline (95% confidence interval [CI]) of UACR by 41% (−0.64/0.96), while in the placebo group a tiny increase of 2% (−0.13/0.45) was noticed (*p* = 0.019) (Fig. 2). The beneficial effect of GTP on albuminuria was maintained even when we included only patients with diabetes mellitus (DM) type 2 in the analyses, resulting in a 37% reduction vs. a 4% increase (*p* = 0.03) for the GTP and placebo groups, respectively. Notably, 19% of patients in the GTP group and none in the placebo group moved from macroalbuminuria or microalbuminuria to microalbuminuria or normoalbuminuria (*p* = 0.03).

### Secondary endpoints

Median values for changes in 24-hour systolic and diastolic blood pressures, body mass index (BMI), glycated haemoglobin (HbA1c), estimated glomerular filtration rate (eGFR) values, and serum C-reactive protein (CRP) were not significantly different between the two groups (Table 2). However, the addition of GTP to the existing treatment regimen significantly reduced mean serum DKK-1 (*p* = 0.004) and tumor necrosis factor alpha (TNF-α; *p* = 0.003) with no significant change in urinary 8-isoprostane concentrations (Table 3).

### Adverse events

No patients withdrew from the study because of adverse events associated with treatment. One patient developed diarrhea and another had dyspepsia after GTP treatment. One patient reported dizziness after placebo treatment.

### *In vitro* studies

#### Plasma from diabetic patients increased albumin permeability and markers of apoptosis in immortalized human podocytes (iHPs)

**Figure 3:**
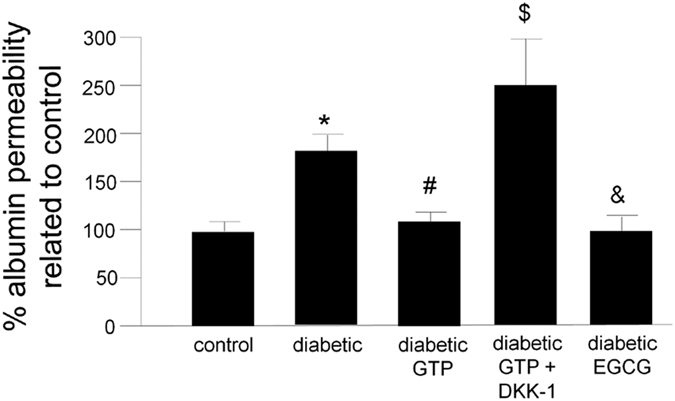
Analysis of the filtration barrier function of the podocyte monolayer by an albumin efflux assay. Differentiated iHPs were incubated with plasma from each healthy controls (n = 3) and plasma from each diabetic subjects before (n = 3) or after 12 weeks treatment with GTP (n = 3) and albumin influx through podocyte monolayer was assessed after 6 hours, as described in methods. Data are presented as mean and vertical bars represent the standard deviation. *P < 0.001 vs. control, ^#^*P* = 0.003 vs. diabetic, ^$^*P* < 0.001 vs. diabetic GTP, ^&^*P* = 0.01 vs. diabetic. GTP, green tea polyphenol; DKK-1, dickkopf 1, EGCG, epigallocatechin gallate.

**Figure 4:**
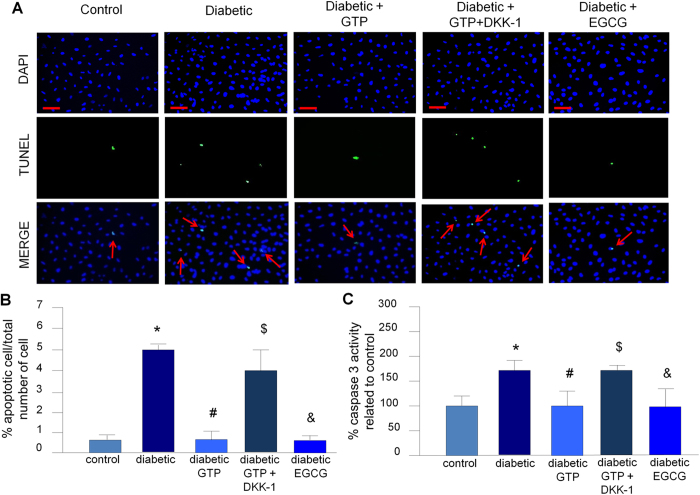
Analysis of podocyte apoptosis. (**A,B**) TUNEL assay, scale bars 50 μm. Bars in B represent the median of three human plasma samples derived from an average of ten fields. Vertical bars represent the standard deviation. *N* = 3 for each condition. *P = 0.01 vs. control, ^#^*P* = 0.01 vs. diabetic, ^$^*P* < 0.001 vs. diabetic GTP, ^&^*P* < 0.001 vs. diabetic. (**C**) Caspase-3 activity. Data are presented as mean ± SD; *N* = 3 for each condition. *P < 0.001 vs. control, ^#^*P* < 0.001 vs. diabetic, ^$^*P* < 0.001 vs. diabetic GTP, ^&^*P* = 0.007 vs. diabetic. GTP, green tea polyphenol; DKK-1, dickkopf 1, EGCG, epigallocatechin gallate.

Treatment of iHPs with plasma from diabetic subjects resulted in increased albumin permeability (*p* < 0.001, Fig. 3) and markers of apoptosis (caspase 3 activity, *p* < 0.001 and terminal deoxynucleotidyl transferase dUTP nick end labeling [TUNEL], *p* = 0.001) (Fig. 4) as compared to podocytes treated with plasma from control subjects. Notably, we did not observe differences in these same parameters when we compared podocytes treated with plasma from control subjects with podocytes treated with plasma from diabetic patients who received GTP (Figs 3 and 4).

#### Addition of DKK-1 to plasma from diabetic subjects who received GTP reversed the protective effect on albumin permeability and podocyte apoptosis markers

To test the possible contribution of WNT pathway activation to increased albumin permeability and podocyte apoptosis markers, we treated plasma from diabetic patients who received GTP with DKK-1, a WNT inhibitor. Interestingly, the protective effect of GTP was eliminated by the addition of DKK-1 (Figs 3 and 4).

#### Addition of EGCG to plasma of diabetic subjects reduced albumin permeability and markers of podocyte apoptosis

Addition of EGCG (436 nM) to plasma from diabetic subjects who did not receive GTP reduced albumin permeability (*p* = 0.01) and markers of podocyte apoptosis (*p* = 0.007) to the levels observed in podocytes treated with plasma from normal subjects (Figs 3 and 4).

## Discussion

To our knowledge, this study is the first to demonstrate the efficacy and safety of adding GTP to maximum doses of ACE inhibitors and/or ARBs in order to reduce residual albuminuria in patients with diabetic nephropathy. Improvement in glomerular barrier selectivity due to reduction in podocyte apoptosis as a result of decreased plasma DKK-1 and WNT pathway activation may be the possible mechanism behind this observed efficacy. It is also possible that inhibition of inflammatory mediators (such as TNF-α) may have contributed to the beneficial effect of GTP^17^. The findings of the current study confirm in a clinical setting our preclinical study that suggested the ability of green tea to reduce albuminuria in diabetic nephropathy, probably by diminishing podocyte apoptosis through activation of the WNT pathway^12^.

Previous studies have demonstrated that plasma DKK-1 is indeed elevated in type 2 diabetic patients in comparison to plasma from normal subjects^18^,^19^. In addition, elevated plasma concentrations of DKK-1 are associated with macrovascular disease in patients with type 2 DM^18^, as well as endothelial dysfunction and platelet activation^19^. Interestingly, plasma concentrations of DKK-1 can be reduced with improved glycemic control and low-dose aspirin treatment^19^. In the present study, we demonstrated that GTP reduces plasma concentrations of DKK-1 independently of its effect on glycemic and blood pressure control.

DKK-1 is a major regulator of the WNT pathway, a group of highly conserved secreted mediators that regulate a wide range of cellular processes, such as proliferation and differentiation, survival, cell fate determination, and migration^20^. Scant but controversial evidence exists for the role of the WNT pathway in podocyte apoptosis. It has been suggested that in experimental DM, high glucose may activate the WNT pathway through stimulation of the transient receptor potential channel 6 (TRPC6), which eventually contributes to podocyte apoptosis^21^. On the contrary, Kato and colleagues have suggested that down-regulation of the WNT pathway in podocytes might in fact enhance apoptosis susceptibility^14^. This last suggestion was confirmed by our recent observations, wherein we demonstrated—both *in vivo* and *in vitro*—that high glucose or blockage of the WNT receptor, LRP6, with DKK-1 or silencing RNA increases glycogen synthase kinase 3 beta (GSK3β), its interaction with p53, and ultimately podocyte apoptosis^12^. GSK3β is involved in WNT pathway regulation^22^. Its activation promotes proteasomal degradation of the transcription factor β-catenin and inhibits cytoplasmic accumulation and subsequent nuclear translocation^22^. Therefore, WNT target genes are not transcripted by β-catenin^22^. We are unaware of any study that has investigated the role of DKK-1/WNT pathway in podocyte apoptosis in human subjects.

Previous research has shown that in cultured podocytes, plasma from diabetic subjects with nephropathy induces apoptosis, as assessed by cleaved caspase 3^23^. Herein, we confirmed that plasma from diabetic patients with nephropathy promotes podocyte apoptosis, as assessed by cleaved caspase 3 and TUNEL assay (Fig. 4), likely due to an increased concentration of plasma DKK-1. This suggestion is supported by our observation that the protective effect of plasma from diabetic patients who received GTP was lost when we treated the podocytes with DKK-1 (Fig. 4). Similar results were also observed in an *in vitro* model of albumin permeability (Fig. 3).

We could not detect a significant correlation of UACR with DKK-1, nor with TNF-α. These findings may be explained by our small sample size, and hence the low power of this study to detect such correlations. However, when compared with placebo, GTP therapy significantly reduced DKK-1 and TNF.

The main limitations of our study and our interpretation of its results are the small sample size and the short duration of the treatment phase. Moreover, in addition to albuminuria, future studies should consider other outcome measures, such as time to renal replacement therapy.

In conclusion, to our knowledge this is the first randomised, double-blind, placebo-controlled clinical trial to demonstrate the possible efficacy and safety of GTP addition to RAS inhibitors in attenuating residual albuminuria in patients with diabetic nephropathy. This observed efficacy may be due to the capacity of GTP to reduce podocyte apoptosis as a result of reduction of DKK-1. Future multicenter randomized trials with larger samples sizes are necessary to confirm the long-term efficacy and safety of adding GTP to RAS inhibitors with the goal of minimizing the progression of diabetic nephropathy.

## Materials and Methods

This randomised, double-blind, placebo-controlled, single-center phase 2 study was conducted in compliance with local and national regulations, Good Clinical Practices guidelines, and Declaration of Helsinki Principles and it was approved by the local Institutional Review Board (Comitê de Ética em Pesquisa, Faculdade de Ciências Médicas, UNICAMP, 18445613.3.0000.5404). All patients signed informed consent before enrollment in the study.

### Patients

Eligible patients, identified during routine visits to the Diabetic Nephropathy Clinic at the University Hospital of the State University of Campinas (UNICAMP), were 18 years of age or older, had been previously diagnosed with DM type 1 or 2, and had persistent micro- or macroalbuminuria (UACR over 30 mg/g as determined by three consecutive urine samples obtained on different days) despite treatment with maximum doses of ACE inhibitors and/or ARBs for at least 8 weeks prior to the screening. Included patients also had HbA1c levels <10%, and regularly used insulin and/or oral glucose lowering agents. Exclusion criteria included autoimmune disease, human immunodeficiency virus (HIV) infection, viral hepatitis, neoplasia, pregnancy and lactation, eGFR below 30 mL/min per 1.73 m^2^ as estimated by the CKD Epidemiology Collaboration (CKD-EPI) equation, chronic urinary tract infection, chronic heart failure of New York Heart Association (NYHA) class III or IV, recent history of coronary artery disease, cerebrovascular accidents, history of alcohol dependency or drug abuse, any psychiatric or neurological conditions preventing mindful consent to the study and/or adherence to the study protocol, and intolerance to green tea.

### Randomization and Study Design

We screened 370 patients with a diagnosis of diabetes. Of these patients, 47 patients met the inclusion criteria and were randomly assigned to two groups: 24 patients were treated with a maximum dose of ACE inhibitors and/or ARBs plus GTP, and 23 patients were treated with a maximum dose of ACE inhibitors and/or ARBs plus placebo. The patients received either four capsules of GTP (tea polyphenol, TP98, MedKoo Bioscience, Chapel Hill, NC 27516-6222, USA) per day, corresponding to 800 mg of EGCG, or placebo for 12 weeks. The percentage of EGCG, epigallocatechin, and epicatechin present in this GTP was confirmed by our laboratory (Supplementary Table 1). The dosage was chosen because it has been shown to be safe and because it might be effective for lowering LDL cholesterol^16^. All drugs and placebo tablets were similar in size, shape, weight, and color. The website Randomization.com (http://www.randomization.com) was used to generate the randomization list. All drug and placebo tablets were prepared by Dermage (Campinas, São Paulo, Brazil), and prepacked bottles were numbered for each patient according to the randomization sequence. All clinical investigators, laboratory personnel, and patients were masked to the treatment assignment. To avoid researcher influence, the randomization list was generated and maintained by trained personnel in a different location from the study.

### Procedures and Outcomes

Patients were evaluated at baseline and then again after 12 weeks. At these two time points, patients provided three samples of first morning urine to determine albuminuria (primary endpoint) and a complete physical examination was performed, including office and 24-hour blood pressure, and BMI. A fasting blood sample was also obtained for determining secondary endpoints including: eGFR, glycemia, HbA1c, lipid profile, CRP, plasma concentration of DKK-1, TNFα, and urinary 8-isoprostane. Because of our recent preclinical observation^12^ showing the potential of green tea to activate the WNT pathway and to reduce albuminuria under diabetic conditions, we post hoc estimated the plasma concentration of DKK-1, a WNT inhibitor, as a secondary outcome. We also post hoc estimated plasma TNF as a marker of inflammation. Patient adherence to the study was evaluated by monthly phone calls and by tablet counts at the end of the study. Adverse events were assessed during the visits and by phone calls.

### Laboratory procedures

Urine samples taken over three consecutive mornings, were used to test for albuminuria at the beginning and the end of the study (Nephelometry, BN II System, Siemens, Germany). Ambulatory blood pressure was monitored over 24 hours (Spacelabs MAPA 90207, Washington, USA) and was measured every 15 minutes during the day and every 30 minutes during the night. Hemoglobin concentration (Sysmex XE-2100, Sysmex Corp., Kobe, Japan), plasma potassium, sodium, creatinine, urea, and lipids (Roche/Hitachi MODULAR P, Roche Lab Systems, Germany) were measured by autoanalyser, CRP was measured by nephelometry (BN ProSpec System, Siemens), and HbA1c was determined using high-performance liquid chromatography (HPLC, Bio-Rad VARIANT II, USA). Plasma DKK1 (R&D, Minneapolis, MN, USA), TNFα (R&D), and urinary 8-isoprostane (ABCAM, Cambridge, MA, USA) were determined using commercially available ELISA kits in accordance with the manufacturer’s instructions.

### Human podocyte culture

Conditionally iHPs (M.A. Saleem, Academic Renal Unit, University of Bristol, Southmead Hospital, Bristol, UK) were provided by Luigi Gnudi (King’s College, London, United Kingdom) and were derived and cultured as reported by Saleem *et al*.^24^. Differentiated podocytes cell passage numbers were between 12 and 17, with batches of cells from the same passage number used for each set of experiments. Replicate experiments were performed (the number of experiments is stated in each case below) and representative results are shown. After overnight serum starvation, differentiated cells were exposed to the treatments for the indicated duration. The concentrations of treatments used in all experiments were chosen after carrying out a thiazolyl blue tetrazolium bromide assay (Supplementary Fig. 1).

### Human podocyte treatments

Starved iHPs were incubated with 4% plasma from each healthy control (*n* = 3) or from each type 2 diabetic patient treated with GTP, obtained before treatment (*n* = 3) or after 12 weeks of treatment with GTP (*n* = 3). Cells were incubated for 6 hours in the presence or absence of a low-density lipoprotein receptor-related protein (LRP) 6 (LRP6) blocker (100 ng/mL DKK-1) (R&D, Minneapolis, MN, USA) or EGCG (436 nM). This concentration of EGCG corresponds to the plasma concentrations obtained after administration of 800 mg of EGCG to healthy controls^25^. These experiments were performed at least twice. Data from the healthy controls and diabetic subjects used in these *in vitro* experiments are presented in Supplementary Table 2. Plasma from diabetic patients and control subjects was obtained at fasting state.

### Caspase-3 activity assay

Caspase-3 activity in iHPs was analyzed using a colorimetric assay^26^.

### TUNEL assay

The TUNEL method was applied to iHPs using a DeadEnd Fluorometric TUNEL System detection kit (Promega, Madison, WI, USA), according to the manufacturer’s instructions. The quantitative analysis of apoptosis in the cultured podocyte cells was carried out by counting the number of positive cells per total number of cells per square millimeter^12^. Ten microscopic images were evaluated for each individual.

### Albumin influx assay

A simple albumin influx assay was used to evaluate the filtration barrier function of the podocyte monolayer as described previously^12^,^27^. Briefly, podocytes (3 × 10^5^) were seeded onto the collagen-coated transwell filters (3 μm pore; Corning, New York, NY) in the top chamber and cultured under differentiating conditions. After 4 days, podocytes were serum-starved overnight and treated for 6 hours with individual plasma samples from normal subjects (*n* = 3) and with plasma from the same diabetic patients before (*n* = 3) or after treatment with GTP (*n* = 3). Cells were washed twice with phosphate buffered saline (PBS) supplemented with 1 mmol/L MgCl_2_ and 1 mmol/L CaCl_2_ to preserve the cadherin-based junctions. The top chamber was then refilled with 0.5 mL of RPMI 1640 and the bottom chamber with 1 mL of RPMI 1640 supplemented with 40 mg/mL of bovine serum albumin and incubated at 37 °C. A small aliquot of media from the top chamber was collected as a single sample 6 hours later and the albumin concentration was determined using a bicinchoninic acid protein assay kit (Sigma).

### Statistical analysis

In accordance with a previous trial of similar design^28^, we calculated that a minimum of 19 patients in each group was necessary to detect a 30% change in UACR (α = 0.05, β = 0.20)^29^,^30^. Data were analyzed using an intention-to-treat principle, defined as participants who met all of the inclusion criteria, met none of the exclusion criteria, had at least one dose of the study drug, and final laboratory assessment data available for analysis. Clinical parameters are presented as medians and interquartile ranges unless otherwise noted. Exploratory data analysis was performed through summary measures of categorical data and descriptive statistics of quantitative data. To compare categorical clinical variables between groups, the chi-square test was applied and, when necessary, Fisher’s exact test. The Mann–Whitney test was performed for numerical comparison of baseline clinical variables between groups and from baseline to the end of the study. The primary outcome for albuminuria is presented as the geometric mean of % change from baseline and two-sided 95% confidence intervals. The within-group geometric mean change (%) is derived by 100x (exp(LS mean change) −1, and the same transformation is applied on the 95% confidence interval for the geometric mean change (%). For the change from baseline in the urinary albumin-to-creatinine ratio (and associated 95% CI), values were back-transformed to provide GTP-to-placebo ratios. *In vitro* data presented as mean ± SD were analyzed with a one-way analysis of variance (ANOVA) followed by Bonferroni test. The significance level adopted for this study was *p* < 0.05. Statistical analyses were conducted using SAS (Statistical Analysis System) software for Windows (version 9.4, SAS Institute Inc., 2002–2008, Cary, NC, USA).

## Additional Information

**How to cite this article**: Borges, C. M. *et al*. The use of green tea polyphenols for treating residual albuminuria in diabetic nephropathy: A double-blind randomised clinical trial. *Sci. Rep.* **6**, 28282; doi: 10.1038/srep28282 (2016).

## Supplementary Material

Supplementary Dataset 1

## Figures and Tables

**Table 1:** Baseline characteristics of patients^.

Characteristic	Green tea polyphenol (*N* = 23)	Placebo (*N* = 24)
Age–yr	63 (60–65)	59 (49–63)
Male sex–no. (%)	11 (47.8)	16 (66.7)
DM type 2–no. (%)	23 (100)	19 (79)
Known duration of DM–yr	16 (12–20)	19 (13–22)
Use of insulin–no. (%)	17 (73.9)	19 (79.2)
Use of statins–no. (%)	19 (82.6)	22 (91.7)
Use of aspirin–no. (%)	17 (73.9)	17 (70.8)
Body-mass index–kg/m^2^	30.6 (27.5–34.7)	32.7 (28.6–35.5)
Waist circumference–cm	107 (99–113)	114 (99–119)
Diabetic retinopathy–no. (%)	19 (82.6)	17 (70.8)
ACEi/ARB/ACEi+ARB–no.	10/12/1	7/14/3
Systolic blood pressure–mmHg		
Office	151 (140–159)	140 (131–160)
24 hour	140 (128–144)	132 (118–139)
Diastolic blood pressure–mmHg		
Office	89 (81–97)	84 (76–99)
24 hour	76 (70–82)	73 (69–79)
Fasting plasma glucose–mg/dL	136 (118–180)	143 (99–194)
Glycated hemoglobin–%	7.7 (7.3–8.3)	8.2 (7.5–9.2)
Serum triglycerides–mg/dL	163 (110–195)	144 (97–207)
Serum total cholesterol–mg/dL	157 (136–169)	156 (147–186)
Serum LDL-C–mg/dL	84 (68–90)	86 (69–91)
Serum HDL-C–mg/dL	40 (33–46)	42 (36–51)
Serum CRP–mg/dL	0.40 (0.08–0.72)	0.23 (0.08–0.47)
eGFR–mL/min per 1.73 m^2^#	55.7 (44.3–71.7)	65.6 (46.2–85.6)
UACR–mg/g&	210 (39–1267)	427 (77–4051)
Micro/Macroalbuminuria–no. (%)¶	16 (70)/7 (30)	10 (42)/14 (58)

Abbreviations: DM, diabetes mellitus; ACEi, angiotensin-converting enzyme inhibitor; ARB, angiotensin receptor blocker; LDL, low-density lipoprotein; HDL, high-density lipoprotein; CRP, C-reactive protein; UACR, urinary albumin-to-creatinine ratio.

^^^Values are median and interquartile ranges unless otherwise noted.

^#^The estimated glomerular filtration rate (eGFR) was calculated using the Chronic Kidney Disease Epidemiology Collaboration formula.

^&^Values are median (range).

^¶^Microalbuminuria, UACR = 30–300 mg/g; macroalbuminuria, UACR >300 mg/g.

**Table 2:** Change from baseline in the two groups.

	GTP (*N* = 21)	Placebo (*N* = 21)	*P* value between groups
Δ 24-hour systolic BP–mmHg	−0.02 (−0.03/0.01)	−0.02 (−0.08/0.04)	0.62
Δ 24-hour diastolic BP–mmHg	−0.01 (−0.06/0.05)	0.01 (−0.02/0.08)	0.35
BMI–kg/m^2^	0.01 (0.00/0.02)	0.00 (−0.01/0.01)	0.32
HbA1c–%	0.01 (−0.06/0.07)	0.02 (−0.04/0.05)	0.86
CRP–mg/dL	0.15 (−0.30/0.40)	0.00 (−0.28/0.60)	0.29
eGFR–mL/min per 1.73 m^2#^	−0.07 (−0.18/0.02)	−0.01 (−0.13/0.09)	0.42

Data are expressed as median of percentage change between the two evaluation points, baseline and 12 weeks (interquartile range).

^#^The estimated glomerular filtration rate (eGFR) was calculated using the Chronic Kidney Disease Epidemiology Collaboration formula.

Abbreviations: GTP, green tea polyphenol; BP, blood pressure; BMI, body-mass index; HbA1c, glycated hemoglobin; CRP, C-reactive protein.

**Table 3:** Median and change from baseline of the two groups.

	GTP	Placebo	*P* Value between groups
**DKK-1 (pg/mL)**			
Median baseline (IQR)	787 (631/948)	839 (495/1063)	0.84
Δ Median after 12 weeks (IQR)	−0.39 (−0.62/−0.26)	0.01 (−0.05/0.13)	<0.001
**TNF-α (pg/mL)**			
Median baseline (IQR)	13.5 (11.3/17.5)	14.5 (11.8/15.1)	0.82
Δ Median after 12 weeks (IQR)	−0.16 (−0.28/−0.06)	0.06 (−0.03/0.15)	<0.001
**8-isoprostane levels (fg/mg creatinine)**			
Median baseline (IQR)	8.9 (7.1/2.5)	7.5 (4.5/15.6)	0.60
Δ Median after 12 weeks (IQR)	0.09 (−0.12/0.42)	0.05 (−0.06/0.33)	0.87

Abbreviations: GTP, green tea polyphenol; DKK-1, Dickkopf WNT pathway inhibitor 1; TNF-α, tumor necrosis factor alpha.
