# Supplementary material for: The use of green tea polyphenols for treating residual albuminuria in diabetic nephropathy: A double-blind randomised clinical trial
Source: Sci Rep. 2016 Jun 20;6:28282. doi: 10.1038/srep28282 (PMC4913255; doi:10.1038/srep28282)

Supplementary Information

**The use of green tea polyphenols for treating residual albuminuria in diabetic nephropathy: a double-blind randomized clinical trial**

# Cynthia M. Borges^1^ MD, Alexandros Papadimitriou^1,2^ PhD, Diego A. Duarte^1^ PhD, Jacqueline M. Lopes de Faria^1^ MD, PhD, José B. Lopes de Faria^1*^ MD, PhD

Table 1: Quantitative characterization of the components of decaffeinated tea polyphenol as determined by high performance liquid cromatography (HPLC).

| Components | Quantity (%) |
| --- | --- |
| Polyphenols | 99.8 |
| Catechins | 78.1 |
| Epigallocatechin | 22.2 |
| DL catechin | 1.5 |
| Epicatechin | 9.3 |
| Epigallocatechin gallate | 41.1 |
| Gallocatechin gallate | 1.7 |
| Epicatechin gallate | 2.1 |
| Caffeine | 0.34 |

**Table 2: Clinical and laboratory features of individuals that provided plasma for podocyte experiments**

|  | **Gender** | **Age**  **(years)** | **Glycemia**  **(mg/dL)** | **AUCR**  **(mg/g Cr)** |
| --- | --- | --- | --- | --- |
| Control |  |  |  |  |
| 1 | F | 55 | 93 | NA |
| 2 | M | 50 | 103 | NA |
| 3 | F | 52 | 84 | NA |
| Diabetic |  |  |  |  |
| 1 | F | 65 | 282 | 80 |
| 2 | F | 68 | 91 | 81 |
| 3 | M | 64 | 134 | 364 |

Figure 1: MTT assay testing cytotoxicity of different dose of DKK-1 in cultured human podocytes. Control (untreated) is considered as 100% viability. All experiments was performed in quadruplicate.


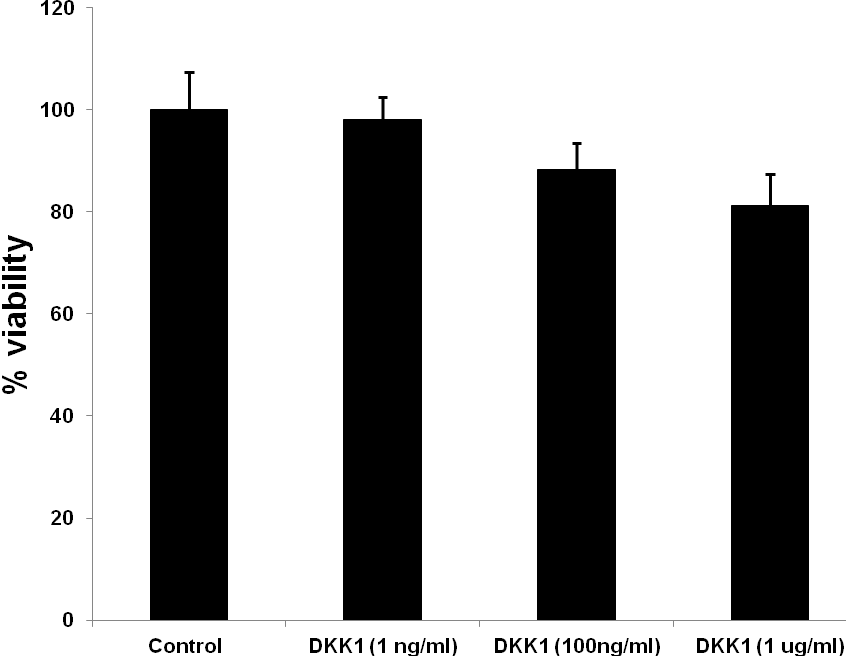

Supplement: Supplementary Dataset 1 [file srep28282-s1.doc]
